# Serum Steroid Ratio Profiles in Prostate Cancer: A New Diagnostic Tool Toward a Personalized Medicine Approach

**DOI:** 10.3389/fendo.2018.00110

**Published:** 2018-04-05

**Authors:** Adriana Albini, Antonino Bruno, Barbara Bassani, Gioacchino D’Ambrosio, Giuseppe Pelosi, Paolo Consonni, Laura Castellani, Matteo Conti, Simone Cristoni, Douglas M. Noonan

**Affiliations:** ^1^IRCCS MultiMedica, Milan, Italy; ^2^Department of Medicine and Surgery, University Milano-Bicocca, Milan, Italy; ^3^Department of Oncology and Hemato-Oncology, University of Milan, Milan, Italy; ^4^I.S.B.—Ion Source & Biotechnologies, Bresso, Italy; ^5^Sant’Orsola Hospital, Bologna, Italy; ^6^Department of Biotechnologies and Life Sciences, University of Insubria, Varese, Italy

**Keywords:** steroids, steroids profile, prostate cancer, algorithm, EU law

## Abstract

**Background:**

Serum steroids are crucial molecules altered in prostate cancer (PCa). Mass spectrometry (MS) is currently the elected technology for the analysis of steroids in diverse biological samples. Steroids have complex biological pathways and stoichiometry and it is important to evaluate their quantitative ratio. MS applications to patient hormone profiling could lead to a diagnostic approach.

**Methods:**

Here, we employed the Surface Activated Chemical Ionization-Electrospray-NIST (SANIST) developed in our laboratories, to obtain quantitative serum steroid ratio relationship profiles with a machine learning Bayesian model to discriminate patients with PCa. The approach is focused on steroid relationship profiles and disease association.

**Results:**

A pilot study on patients affected by PCa, benign prostate hypertrophy (BPH), and control subjects [prostate-specific antigen (PSA) lower than 2.5 ng/mL] was done in order to investigate the classification performance of the SANIST platform. The steroid profiles of 71 serum samples (31 controls, 20 patients with PCa and 20 subjects with benign prostate hyperplasia) were evaluated. The levels of 10 steroids were quantitated on the SANIST platform: Aldosterone, Corticosterone, Cortisol, 11-deoxycortisol, Androstenedione, Testosterone, dehydroepiandrosterone, dehydroepiandrosterone sulfate (DHEAS), 17-OH-Progesterone and Progesterone. We performed both traditional and a machine learning analysis.

**Conclusion:**

We show that the machine learning approach based on the steroid relationships developed here was much more accurate than the PSA, DHEAS, and direct absolute value match method in separating the PCa, BPH and control subjects, increasing the sensitivity to 90% and specificity to 84%. This technology, if applied in the future to a larger number of samples will be able to detect the individual enzymatic disequilibrium associated with the steroid ratio and correlate it with the disease. This learning machine approach could be valid in a personalized medicine setting.

## Introduction

Hormonal physiology plays an important role in the function of the prostate. Development, growth, and functions of this gland depend on the actions of testosterone and its metabolite dihydrotestosterone (DHT), which enable the growth and proliferation of the glandular component of the prostate through binding and activation of androgen receptor (AR) within the cytoplasm of prostatic epithelial cells (1). In contrast to the chemical and virus-induced tumors, the hormone-related cancers shared a quite different mechanism of carcinogenesis: hormones, both endogenous and exogenous, drive cell proliferation, increasing the number of cell divisions and the opportunity for random genetic errors (2–4). In some cases, hormones can be metabolized to genotoxic agents by enzymes, which can form adducts with DNA and lead to mutations (5). The actions of androgen hormones are very controversial in prostatic hyperplasia [benign prostate hypertrophy (BPH)] and prostate cancer (PCa). Several studies showed that the activation of AR results in proliferative growth of prostatic epithelium, favoring the transition from prostatic hyperplasia (BPH) to PCa (4, 6, 7). However, lower levels of testosterone and DHT are associated with BPH and PCa (8–11). As men age, the free serum testosterone declines by 2–3% approximately annually (12); yet, there is increase in the incidence of BPH, and longevity increases the number at risk. Also, men who have high body mass index have larger prostates but lower testosterone levels (13). There is a potential explanation of lower levels of testosterone and DHT with BPH, as explained through a murine model where the authors blocked AR activation in the luminal cells, leading to inflammation of the prostate gland in these animals (14). Inflammation and subsequent angiogenesis lead to PCa progression (15), and we identified a fragment of complement C3f in low prostate-specific antigen (PSA) samples (16). PSA is a specific prostate marker, but only marginally influences mortality from PCa, and a recent study of the available PCa biomarkers suggested more candidate biomarkers (17).

Most of the studies measuring testosterone and DHT (cited above) used immunoenzymatic (ELISA) and radioimmuno-assay (RIA) to measure total testosterone, then applied Vermeulen’s formula (based on sex hormone-binding globulin and albumin) to calculate free testosterone. Analysis of testosterone in serum is quite complicated, especially in ELISA and RIA conditions, where the low amount and enzymatic cross-reactions seriously affect their quantitation (18). Recently, mass spectrometry (MS) has been elected as the reference technology for steroid dosage (19–23) since the sensitivity and specificity of this technology avoids the quantification problems in ELISA and RIA conditions. Steroids dosage also represents a crucial task for the diagnosis of different diseases (22, 24–27), such congenital adrenal hyperplasia (28), cardiovascular (22), and other degenerative diseases (24, 26, 27).

Liquid chromatography tandem MS operating in multiple reaction monitoring conditions (LC-MS/MS-MRM) is actually the most employed technology for steroids analyses (23, 29). Different methods have been developed in both electrospray (ESI) (30) and Atmospheric Pressure Ionization conditions (APCI) (31) combined to triple quadrupole (QQQ) as mass analyzer (32). QQQ has gained the consensus of clinical institutes mainly due to its higher quantitation accuracy in target analysis with respect to other analyzers (29, 32).

In addition to quantification of steroids by LC-MS/MS, other investigations show that the clinical information correlated with the disease is not only contained in the absolute steroid quantitation, but rather in their ratios (33, 34). This reflects their synthesis starting from the precursor cholesterol and the enzymes involved (Figure S1 in Supplementary Material). For example, a deficit in 21-hydroxylase leads to a decrease in mineralocorticoids and glucocorticoids and to an increase of androgens and testosterone (4). Thus, the ratio can be an informative and complementary diagnostic tool.

Surface Activated Chemical Ionization-Electrospray-NIST Bayesian model database search (SANIST) platform has been recently proposed by us as a powerful tool for personalized medicine (28, 35). Basically, it is an open platform based on three core points:

(a) Standardized kits for sample preparation to maximize the inter-laboratory data reproducibility.
(b) Use of SACI/ESI ionization source to reduce the instrument chemical noise and increase the number of detectable analytes.
(c) The SANIST data elaboration technology that is based on an open model: different applications are progressively added depending to the analysis target.

Concerning the point (c) SANIST has been currently implemented with two applications:

(a) An application to be used for biomarker discovery, able to classify samples and diagnose diseases on the basis of Bayesian machine learning approach (35). This technology makes possible to consider the individual variability and correct the diagnose bias obtained using fixed biomarkers (36, 37).
(b) An application to identify the analytes in conformity with the guidelines suggested by the European Community (EU directive 2002/657/EC) (35).

We describe here the development and application of a new SANIST platform to quantify steroids for diagnostic purposes going toward a personalized medicine approach. In detail, 10 steroids [aldosterone, corticosterone, cortisol, 11-deoxycortisol, androstenedione, testosterone, dehydroepiandrosterone (DHEA), dehydroepiandrosterone sulfate (DHEAS), 17-OH-progesterone and progesterone] have been quantitatively detected by means of SANIST technology and their profile has been analyzed for PCa diagnosis. The profiles of 71 serum samples (31 controls, 20 patients with PCa, and 20 subjects with benign prostate hyperplasia) were evaluated. The ability of the steroids profiling to discriminate among the different groups was tested both considering the individual percent concentration and the relative ratio relationships, showing that the predictive performances are better when using the relative relationship conditions.

## Materials and Methods

### Chemicals

A CE-IVD certified and standardized kit for steroids analysis was purchased from ISBN (Varese, Italy). The kit contains LC mobile phases (phases A and B), the sample precipitation reagent E and the sample resuspension reagent D. Aldosterone, Corticosterone, Cortisol, 11-deoxycortisol, Androstenedione, Testosterone, DHEA, DHEAS, 17-OH-Progesterone and Progesterone standards were purchased from (ISBN laboratory, Varese, Italy) together with their internal standard (Aldosterone d_8_, Androstenedione ^13^C_3_, Corticosterone d_4_, Cortisol d_4_, 11-Deoxycortisol d_5_, DHEA d_6_, Progesterone ^13^C_3_, 17-OH-Progesterone ^13^C_3_, DHEAS d_6_, Testosterone ^13^C_3_). Analyte controls at defined concentrations were purchased from (ISBN laboratory, Varese, Italy). Table S1 in Supplementary Material reports the internal standard concentrations while Table S2 in Supplementary Material has the known calibrate analyte concentrations and the calculated concentrations of both known and unknown samples.

### Sample Selection, Collection, and Preparation

Samples were selected among 50-year-old male patients being subjected to a prostate biopsy for PCa diagnosis at the Urology Unit of the MultiMedica Castellanza, Varese, Italy, without considering those affected by autoimmune diseases, hypersensitivities, and other immune-mediated physical states. Multiple serum PSA analyses were performed on patients along with digital rectal exam and ultrasound. Many different clinical parameters were used to classify patients among which the histological analysis of the biopsy, total PSA, free-PSA, PSA-velocity (changes in PSA levels over time), ultrasound and rectal analysis. Controls included healthy subjects undergoing PSA dosage at our Urology unit that had a PSA lower than 2.5 ng/mL (28 patients) or less than 4 ng/mL and free PSA greater than 15% (3 patients). The study was approved by the institutional review board ethics committee (Protocol N 10 2 10/2011) and, according to the Helsinki Declaration of 1975 as revised in 2013, informed consent was provided by all patients. 71 serum samples (31 controls, 20 patients with PCa, and 20 subjects with benign prostate hyperplasia) were collected (sample number, age, PSA, AR positivity, and Gleason score are shown in Table 1). Blood samples were treated to obtain sera: they were collected in siliconized tubes, left to coagulate for 30 min and then subjected to centrifuge for 30 min at 3,000 rpm at 4°C to reduce variability through the collection of the samples (38). 1.8-mL cryotube vials (Thermo Fisher Scientific, Rodano, Milan, Italy) were used to collect sera and store them at −80°C until the time of analysis. 10 µL of steroids isotopically labeled internal standard at known concentration (Table S1 in Supplementary Material) were added to 290 µL of serum to normalize them. 600 µL of precipitating reagent E were added to each sample. They were then centrifuged for 1 min at 13,000 × *g*/min using a (Biospin, Eppendorf, Germany). 700 µL of the supernatant were collected in another Eppendorf tube and dried with nitrogen gas. The dried pellet was resuspended using 70 µL of reagent D. The analyte calibrants (Table S2 in Supplementary Material) were treated with the same procedure employed for the sample preparation. Quantitative analysis stability was checked by inserting standard controls at different known concentrations enclosed in the kit employed for the dosage (Table S1 in Supplementary Material). Limit of detection (LOD) and quantitation (LOQ) were calculated using the standard calibration curves (7 points).

**Table 1:** Clinical characteristics of the (a) patients, (b) Benign prostate hypertrophy, and (c) controls subjects.

1A. Patients									

Prostate cancer patients							Classification based on		

ID	Age	Gleason score	Androgen receptor (%)	Total prostate specific antigen (PSA)	Dehydroepiandrosterone sulfate (DHEAS)	Relationship average ratio discriminating coefficient (relative value)	PSA^a^	DHEAS^b^	Relationship average ratio discriminating coefficient (relative value)
A0001	58	7 (3 + 4)	>95	6.00	1,144.79	44.5	Benign prostate hypertrophy (BPH)/HC	BPH/HC	PCa
A0003	78	8 (4 + 4)	>95	43.00	191.792	49.3	PCa	PCa	PCa
A0004	66	7 (3 + 4)	>95	8.00	591.168	38.7	PCa	PCa	PCa
A0005	68	7 (3 + 4)	>95	9.50	638.852	49.8	PCa	PCa	PCa
A0006	76	6 (3 + 3)	>95	7.30	385.659	42.2	BPH/HC	PCa	PCa
A0007	82	7 (4 + 3)	>95	78.00	2,106.52	42.7	PCa	BPH/HC	PCa
A0008	74	7 (3 + 4)	>95	5.29	1,490.21	51.1	BPH/HC	BPH/HC	PCa
A0010	70	7 (4 + 3)	>95	400.00	234.102	52.9	PCa	PCa	PCa
A0011	80	6 (3 + 3)	>95	11.00	247.311	47.6	PCa	PCa	PCa
A0012	69	6 (3 + 3)	>95	10.00	430.209	49.3	PCa	PCa	PCa
A0014	70	6 (3 + 3)	>95	12.00	903.685	40.9	PCa	BPH/HC	PCa
A0015	78	7 (3 + 4)	>95	4.90	294.322	50.2	BPH/HC	PCa	PCa
A0017	72	6 (3 + 3)	>95	12.00	1,286.50	52.0	PCa	BPH/HC	PCa
A0018	69	7 (3 + 4)	>95	ND	1,423.28	5.0		BPH/HC	HC
A0020	61	7 (4 + 3)	>95	5.52	553.783	8.0	BPH/HC	PCa	HC
A0021	75	6 (3 + 3)	>95	5.50	519.083	43.1	BPH/HC	PCa	PCa
A0022	65	7 (3 + 4)	>95	21.00	1,209.71	47.1	PCa	BPH/HC	PCa
A0023	56	7 (3 + 4)	>95	6.29	977.112	47.6	BPH/HC	BPH/HC	PCa
A0024	57	9 (4 + 5)	>95	12.00	1,142.33	51.6	PCa	BPH/HC	PCa
A0025	72	7 (3 + 4)	>95	8.70	168.51	44.9	PCa	PCa	PCa

**1B. Benign prostate hypertrophy**									

**Benign prostate hypertrophy**							**Classification based on**		

**ID**	**Age**	**Gleason score**	**Androgen receptor**	**Total PSA**	**DHEAS**	**Relationship average ratio discriminating coefficient (relative value)**	**PSA^a^**	**DHEAS^b^**	**Relationship average ratio discriminating coefficient (relative value)**

C0001	57		>95	5.20	1,443.20	22.6	BPH/HC	BPH/HC	BPH
C0002	64		>95	17.00	947.786	19.6	PCa	BPH/HC	BPH
C0003	65		>95	7.27	1,158.89	21.6	BPH/HC	BPH/HC	BPH
C0004	75		>95	9.12	317.604	26.8	PCa	PCa	BPH
C0005	64		>95	11.00	1,550.66	22.8	PCa	BPH/HC	BPH
C0007	62		>95	5.45	1,590.28	44.3	BPH/HC	BPH/HC	PCa
C0008	66		>95	4.68	684.52	48.9	BPH/HC	PCa	PCa
C0009	61		>95	5.40	955.398	23.5	BPH/HC	BPH/HC	BPH
C0010	63		>95	2.50	1,508.79	18.7	BPH/HC	BPH/HC	BPH
C0012	59		>95	5.80	2,363.96	47.4	BPH/HC	BPH/HC	PCa
C0014	60		>95	6.00	2,216.43	50.3	BPH/HC	BPH/HC	PCa
C0016	54		>95	5.50	1,622.97	23.5	BPH/HC	BPH/HC	BPH
C0017	58		>95	6.60	584.228	18.9	BPH/HC	PCa	BPH
C0018	58		>95	12.94	600.57	18.9	PCa	PCa	BPH
C0019	52		>95	10.33	1,552.22	20.3	PCa	BPH/HC	BPH
C0020	50		>95	3.54	899.207	23.9	BPH/HC	BPH/HC	BPH
C0021	69		>95	6.00	901.446	26.4	BPH/HC	BPH/HC	BPH
C0022	63		>95	6.00	1,300.37	21.4	BPH/HC	BPH/HC	BPH
C0023	52		>95	5.44	1,844.59	21.9	BPH/HC	BPH/HC	BPH
C0024	62		>95	8.94	911.52	24.4	PCa	BPH/HC	BPH

**1C. Controls subjects**									

**Healthy controls**					**Classification based on**				

**ID**	**Age**	**Total PSA**	**DHEAS**	**Relationship average ratio discriminating coefficient (relative value)**	**PSA^a^**	**DHEAS^b^**	**Relationship average ratio discriminating coefficient (relative value)**		

HC0001	79	1.11	907.043	5.3		BPH/HC	HC		
HC0002	54	0.5	1,672.22	5.3		BPH/HC	HC		
HC0003	72	1.12	538.336	4.5		PCa	HC		
HC0004	84	ND	298.8	6.4		PCa	HC		
HC0005	64	0.45	1,512.15	49.5		BPH/HC	PCa		
HC0006	54	1.66	531.844	47.4		PCa	PCa		
HC0007	60	1.91	1,853.55	6.0		BPH/HC	HC		
HC0008	50	0.24	1,848.17	5.7		BPH/HC	HC		
HC0009	83	1.23	1,680.05	48.6		BPH/HC	PCa		
HC0010	54	0.33	2,534.77	5.8		BPH/HC	HC		
HC0011	66	1.13	960.77	6.2		BPH/HC	HC		
HC0012	57	1.2	1,440.51	5.6		BPH/HC	HC		
HC0013	73	0.5	1,167.40	5.4		BPH/HC	HC		
HC0014	78	2.53	673.551	49.7		PCa	PCa		
HC0015	70	0.43	602.138	6.1		PCa	HC		
HC0016	65	0.43	1,438.50	5.6		BPH/HC	HC		
HC0017	59	0.24	844.584	5.8		PCa	HC		
HC0018	65	1.94	1,678.04	4.6		BPH/HC	HC		
HC0019	58	1.85	1,440.07	6.3		BPH/HC	HC		
HC0020	67	2.81	1,377.38	47.6		BPH/HC	PCa		
HC0021	55	3.16	1,419.92	4.3		BPH/HC	HC		
HC0022	66	1.79	530.501	4.5		PCa	HC		
HC0023	72	1.99	541.246	5.1		PCa	HC		
HC0024	71	1.21	534.53	48.7		PCa	PCa		
HC0025	68	1.1	627.21	6.1		PCa	HC		
HC0026	82	0.71	1,408.50	6.1		BPH/HC	HC		
HC0027	60	0.63	1,988.76	6.1		BPH/HC	HC		
HC0028	54	1.22	193.359	4.6		PCa	HC		
HC0029	54	0.42	1,037.78	6.0		BPH/HC	HC		
HC0030	79	1.46	430.433	4.8		PCa	HC		
HC0031	84	1.49	1,128.00	4.7		BPH/HC	HC		

*^a^Based on the lower limit of the 95 CI of the geometric mean of PCa patients (7.385)*.

*^b^Based on the upper limit of the 95 CI of the geometric mean of PCa patients (888.7)*.

### Chromatography

To study the selected analytes, a NeXera UPLC liquid chromatography apparatus (Shimatzu, San Jose, CA, USA) was employed. A Hypersylgold C18 2.1 mm × 50 mm 1.8 µm column was used. The ISBN kit has two mobile phases that were employed (Phases A and B). The LC apparatus worked in a binary gradient: 5% of B was kept for 0.5 min, after 3.5 min, B was increased to 45%, after other 8 min, B was raised to 65% and in 0.1 min to 100%. This percent was maintained for 1.9 min, then, 5% of B was achieved in 0.1 min and the column equilibrium was re-established reaching the starting conditions for 2.9 min. The total time of the analysis was 17 min. A 0.55 mL/min chromatographic flow was employed with a 20 µL injection volume.

### Mass Spectrometry

Molecule assessments were achieved using 120 series triple quadrupole (PerkinElmer, USA) operating in SANIST mode. Heated SACI-ESI capillary had a 1,500 V voltage, SACI-ESI surface had a 47 V voltage, dry gas was kept to 2 L/min, 80 psi Nebulizer, and 40°C temperature were set. Collision-induced dissociation conditions were established for tandem MS experiments using Ar as the collision gas. The first quadrupole had an isolation window of ±0.3 *m/z* and the second one of 0.1 *m/z*.

### Spectra Stability Evaluation

Chromatographic peak spectral average was performed to obtain the higher spectrum quality. Only signal intensities higher than 10^3^ counts/s were considered to improve the spectrum quality.

### General Normalization

As a normalization factor, since the concentrations of cortisol and DHEAS were far above any of the other steroid values, they were reduced by dividing 10 and 100 times, respectively.

### SANIST Data Elaboration Platform

The disease classification among controls, PCa, and subjects with benign prostate hyperplasia were obtained using two approaches: (a) singular absolute concentration match and (b) steroid ratio relationship match (Figure S2 in Supplementary Material). The SANIST tool acquires the selected potential steroid quantitation value and calculates the different combinatorial concentration ratio values. The steroids concentration ratio relationships are processed with the novel Bayesian data analysis-based SANIST mathematical model. SANIST data elaboration system calculates the probability that the detected steroid profile was associated to known disease (e.g., PCa). Biomarker profiles of serum from biopsy positive (PCa) and negative (BPH) subjects were analyzed and inserted to previously instruct the system. The probability in which an event occurs is determined by the Bayes’ theorem. Based on this interpretation, Bayes’ theorem is defined as the relationship between P(A) (probability that an acquired profile matches a PCa patient), P(B) (probability that a “fingerprint” biomarker profile corresponds to a biopsy negative subject), P(A | B) and P(B | A) for any events considering A and B in the same event space. In our case A and B indicate the different steroid concentration ratios. Following the Bayesian interpretation, probability, or uncertainty measures the confidence that something is true. Under this theory, Bayes’ theorem relates uncertainty before and after observing proof. The initial uncertainty in A is the prior, P(A). The uncertainty having accounted for evidence B is the posterior, P(A | B). The degree of support B provides for A is represented by the P(B | A)/P(B) ratio. Formula I shows the relationship among the probabilistic function:

(I) $$\text{P}(\text{A|B})\text{=}\frac{\text{P}(\text{B|A})\text{P}(\text{A})}{\text{P}(\text{B})}$$

We introduced a new coefficient in formula (I) that gives formula (II):

(II) $$\text{P}(\text{A|B})\text{=}\frac{\text{P}(\text{B|A})\text{P}(\text{A})}{\text{P}(\text{B})}*C(mz,rt,i)$$

The correction factor *C* (*mz, rt, i*) enables to progressively rise the disease classification accuracy, on the basis of the number of uncorrected sample classification. In our case, the correct classification of the samples as PCa biopsy positive or negative with the SANIST tool. At least 10,000 sequential classification steps are required to reach this result. It is possible to evaluate the probability that the data observed in the serum are correctly classifiable with the theorem considering the steroid levels and different combinatorial concentration ratios.

We used two models for classification of the individual patient profiles:

(a) Singular absolute concentration match. In this model, a vector database containing the analyte *m*/*z* ratio vs the singular analytes concentration and a percent value is obtained. The control, PCa, or benign prostate hyperplasia samples were classified, using the SANIST algorithm (35), by matching their concentration vector again the machine learning database excluding the identity match.
(b) Steroids ratio relationships match. In this model, all the ratios of the concentration of the analyzed steroids are obtained and a vector of ratio index [*R*(*i*)] is obtained for each sample. The vector machine learning database is obtained by classifying each vector as a control, patient with PCa, or with benign prostate hyperplasia. Each subject was classified, using the SANIST algorithm (39), by similarity of its ratio vector versus the database by excluding the identity match.

### SANIST Application for Steroids Relationship Calculation Spectral Matching Algorithm

SANIST proprietary data elaboration platform configuration has shown in a previous publication (35). SANIST application converts the sample amount in ratio index using the formula (1):

(1) R(i,k)(k=1 to (n−1))[(i=(k+1) to (n))]=C(k)/C(i+1)

where *n* is the total number of analyte, *i* is an analyte counter, and *R* is the analyte concentration ratio given by the *C* concentration factor. The formula (1) is repeated for (*n* − 1) cycles so to calculate a number or *R*(*i*) ratio following the *k* index. The obtained ratios are employed in order to obtain a comparison vector with the (*k*;*i*) vs *R*(*i*;*k*). The number of *R* ratios depends on the number of analytes (*n*) and is given by the Eq. 2.

(2) N(R)((k=1 to (​n−1))=Sum (n−i)

### SANIST Application for Steroid Singular Absolute Concentration Match

Classical data comparison was obtained by calculating the comparison vector considering the *m*/*z* vs C normalized (Cn) data. A *m*/*z* virtual parameter having a value of 1,000 was inserted in the vector as normalizing factor and its *C* value (Cv) was given by the sum of all the concentration parameters (see Eq. 3):

(3) Cv=Sum (i=1 to n) (C i)

The Cn normalized data is calculated as a relative concentration with respect to Cv (Eq. 4):

(4) Cn=(Ci/Cv)∗100

The final comparison vector is so given by *m*/*z* vs Cn pairs.

## Results and Discussion

**Figure 1:**
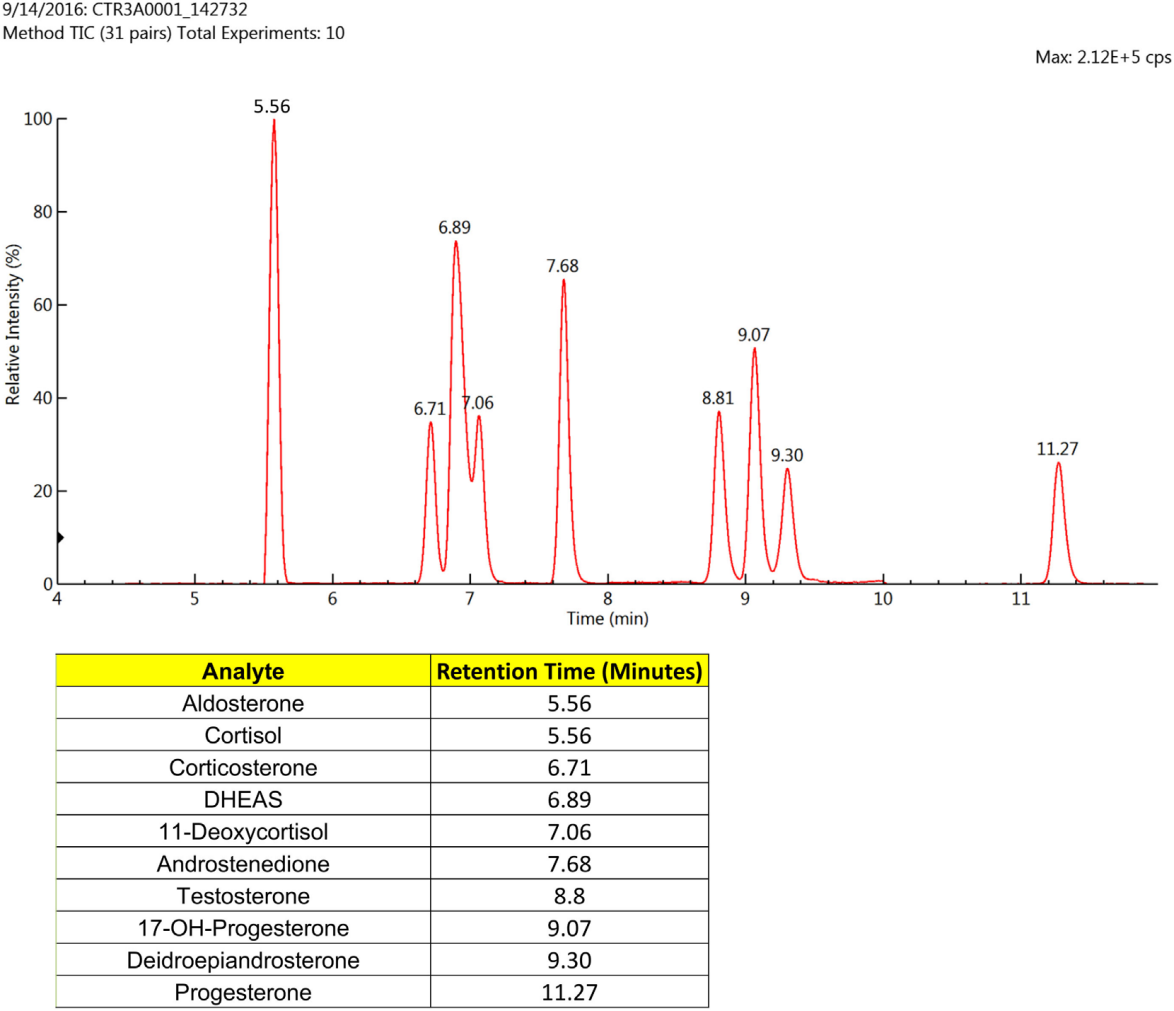
Liquid chromatographic (LC) profiles obtained using the standards for the 10 selected steroids (20 µL) that were subjected to mass spectrometry (MS)/MS-MRM. Only two of the analytes eluted at the same time, but these were easily distinguished by the MS/MS-MRM.

The LC-MS/MS-MRM data acquisition was optimized by monitoring the transitions reported in Table 2. Figure 1 reports the chromatographic profiles obtained by analyzing the 10 selected steroids injecting 20 µL of the calibration 7 standard solution (see Table S2 in Supplementary Material). Aldosterone and Cortisol are the only co-eluting steroids. Their retention time is 5.56 min. However, they are discriminated based on of their *m*/*z* parent ion difference (*m*/*z* 359.2 for aldosterone and *m*/*z* 363.1 for cortisol) and selective MRM transition (*m*/*z* 359.2—>189.1 and 359.2—>331.3 for aldosterone; *m*/*z* 363.1—>121, and 363.1—>115.1 for cortisol). DHEA and DHEAS exhibit the same parent ion, due to the fact that DHEAS loses its sulfate groups in the source. The discrimination between the two compounds occurs on the basis of LC retention time (9.30 min for DHEA and 6.89 min for DHEAS).

**Table 2:** Table reporting the mass spectrometry (MS)/MS MRM analyte transitions.

Steroids	Parent ion	Fragment ion
Aldosterone 1	359.2	189.1
Aldosterone 2	359.2	331.3
Corticosterone 1	347.1	121.1
Corticosterone 2	347.1	97.1
Cortisol 1	363.1	115.1
Cortisol 2	363.1	121
11-deoxycortisol 1	347.11	97
11-deoxycortisol 2	347.11	109
Androstenedione 1	287.1	97
Androstenedione 2	287.1	109
Testosterone 1	289.1	97
Testosterone 2	289.1	109
DHEA 1	271.2	213.1
DHEA 2	271.2	253.2
DHEAS 1	271.2	197.1
DHEAS 2	271.2	213.2
17-OH-Progesterone 1	331	97
17-OH-Progesterone 2	331	109
Progesterone 1	315.1	97
Progesterone 2	315.1	109

Stability and performance of the method mainly in terms of matrix effect, LOD, LOQ, and linearity range were tested. Matrix effect was evaluated both based on internal standards stability and on precision and accuracy parameters (Table 3). Internal standard inter-analysis variation was lower than 5% while intra-day precision and accuracy error were lower than 6 and 8%, respectively. Even interday data show good reproducibility with precision and accuracy error lower than 8 and 9%, respectively. Table 3 reports also the LOD, LOQ, and linearity range for each analyte. The LOD are among 0.01 and 1 ng/mL depending by the analytes while LOQ among 0.05 and 5 ng/mL. Linearity range covers four orders of magnitude for each analyte.

**Table 3:** Precision and accuracy error %, limit of detection (LOD), limit of quantitation (LOQ), and linearity range for each steroid.

Steroids	IS % Variation %	Intraday precision %	Intraday accuracy %	Interday precision %	Interaday accuracy %	LOD	LOQ	Linearity range
11-deoxycortisol	4	5	7	7	8	0.01	0.05	0.05–1,000
17-OH-progesterone	3	5	6	6	8	0.01	0.05	0.05–1,000
Aldosterone	3	3	6	7	8	0.02	0.05	0.05–1,000
Andro-stenedione	4	4	7	7	8	0.05	0.1	0.1–2,000
Corticosterone	4	4	5	6	7	0.05	0.1	0.1–2,000
Cortisol	3	3	4	6	7	1	5	5–7,000
Dehydroepiandrosterone	3	5	6	7	8	0.1	1	1–5,000
Dehydroepiandrosterone sulfate	4	5	7	7	7	1	10	10–7,000
Progesterone	3	4	7	6	7	0.01	0.05	0.05–1,000
Testosterone	3	5	6	7	8	0.01	0.05	0.05–1,000

After verifying the method stability, patient samples were analyzed. The absolute quantitation values for each steroid are reported in Table S2 in Supplementary Material together with the quantitation curves. The steroids concentrations are in different ranges based on the analyzed molecules, which are given for each patient in Table S2 in Supplementary Material. In particular, 11-deoxycortisol, 17-Oh-progesterone, aldosterone, androstenedione, corticosterone, DHEA, progesterone, testosterone concentration is under 50 ng/mL, while DHEAS and cortisol are much higher, in some cases can be over 1,000 ng/mL.

Since the PSA levels were used to determine the control group, this would introduce a bias into the system for determining the performance of PSA. Using PSA for the BPH and the PCa patients, the PSA receiver-operating characteristic (ROC) curve was calculated and area under the curve was 0.7118. We determined a threshold based on the lower limit of the 95 confidence intervals of the geometric mean of the PSA of the PCa patients, lead to a sensitivity (true positives) of 63% and specificity (true negatives) of 59%. The PSA, Gleason score, and AR receptor data of the cancer patients, benign prostate hypertrophy and control subjects are reported in Table 1A–C, respectively.

**Figure 2:**
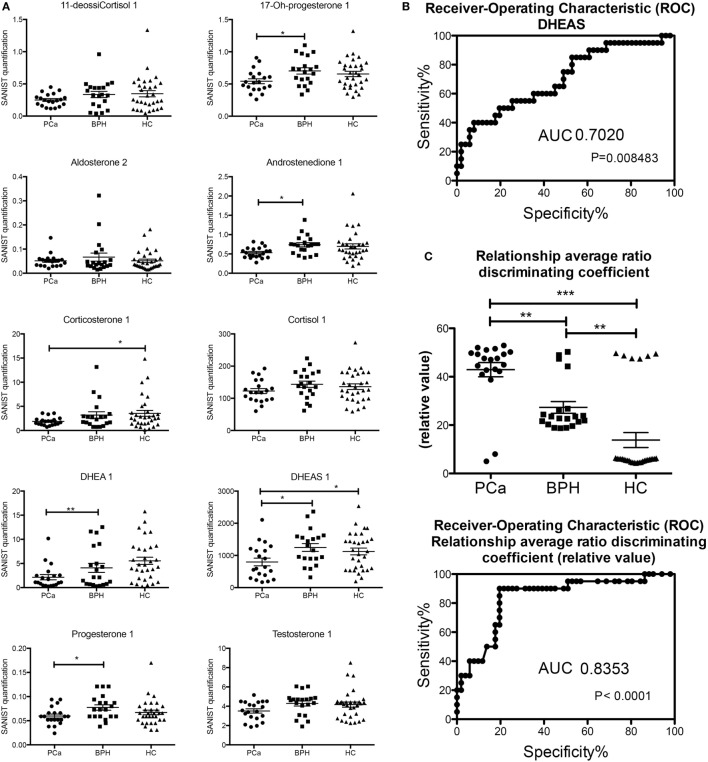
**(A)** Classic analysis of absolute concentrations of the steroids. **(B)** Receiver-operating characteristic (ROC) curve of dehydroepiandrosterone sulfate based on the classification in Table 1. **(C)** Analysis of the output of the machine learning (based on the classification in Table 1) and ROC of the values obtained (*p < 0.05; **p < 0.01; *p* < 0.001 one way ANOVA, Graphpad Prism 5).

Most of the steroids were lower in the PCa patients than in the controls (both healthy and BPH) and are shown graphically in Figure 2. This is in keeping with the lower levels of free testosterone that are associated with “progression” or “reclassification” of PCa (9, 11). However, these data do not coincide with the lower levels of free testosterone in patients with BPH (8, 10) and the inflammatory state with the lack of AR in luminal cells in murine models (14). These studies used RIA conditions, which could affect the level of accuracy. Since more and more clinical studies will be using LC/MS-MS for analyzing androgen levels, our data will be possibly confirmed. Both the enzymes that synthesize androgens (40, 41) and the breakdown of androgens (42, 43) are present in PCa cells and may affect circulating androgen and steroid levels.

**Figure 3:**
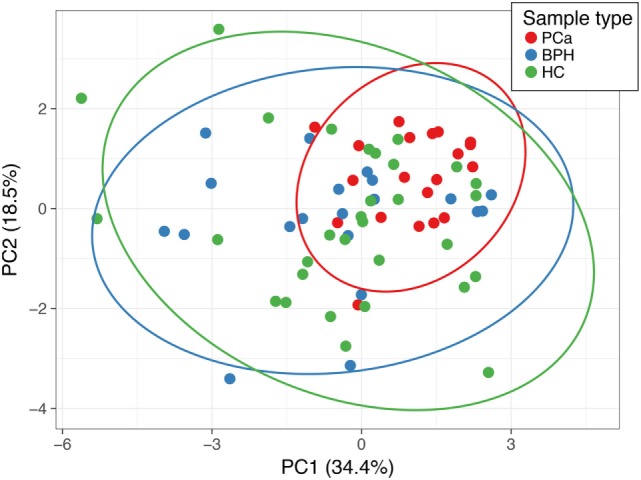
Principal component analysis (PCA) of the absolute (normalized) concentrations of all the steroids. Points labeled in red are PCa patients, in blue are the benign prostate hypertrophy patients, green are the healthy controls. Principal component (PC) 1 and PC 2 explain 34.4 and 18.5% of the total variance, respectively. The prediction ellipses are such that with a probability 0.95, a new observation from the same group will fall inside the ellipse.

There were several statistically significant differences between the hormones that separated the patients with prostate carcinoma versus those of benign prostate hyperplasia and controls. The only hormonal value separating statistically all three cases was the one of DHEAS. We determined a threshold based on the upper limit of the 95 confidence intervals of the geometric mean of the DHEAS of the PCa patients (Table 1). This identified 11/20 (55%) of the PCa patients, 16/20 (80%) of the BPH patients, and 19/31 (62%) of the healthy controls. The ROC curve was calculated and area under the curve (separating the PCa from BPH and HC) was 0.702 (Figure 2). The sensitivity was 55% and the specificity was 59%. We also performed a Principal Component Analysis (PCA) (44) on the steroid absolute values (Figure 3). PCA is a method where a multivariate data set is linearly transformed into a set of uncorrelated variables that are ordered in descending manner, the first few components that often explain large amount of the variation (44). BPH patients and controls overlapped extensively (Figure 3). The vast majority of the PCa patients (Figure 3) were grouped into a restricted subset of the area of the BPH patients and controls.

We then proceeded in calculating the comparison vector employed to build the progressively updated supervised database that will be used for sample classification on the basis of individual steroid enzymatic profiles. When similarity vectors are calculated following the SANIST algorithm (39), the cortisol and DHEAS exhibits a high weight on the calculation function and the other concentration parameters area weakly considered by the discrimination function. To avoid this problem, we “normalized” the weight of the cortisol and DHEAS by dividing their value by 10 and 100, respectively. All the samples were subjected to this normalization model.

**Figure 4:**
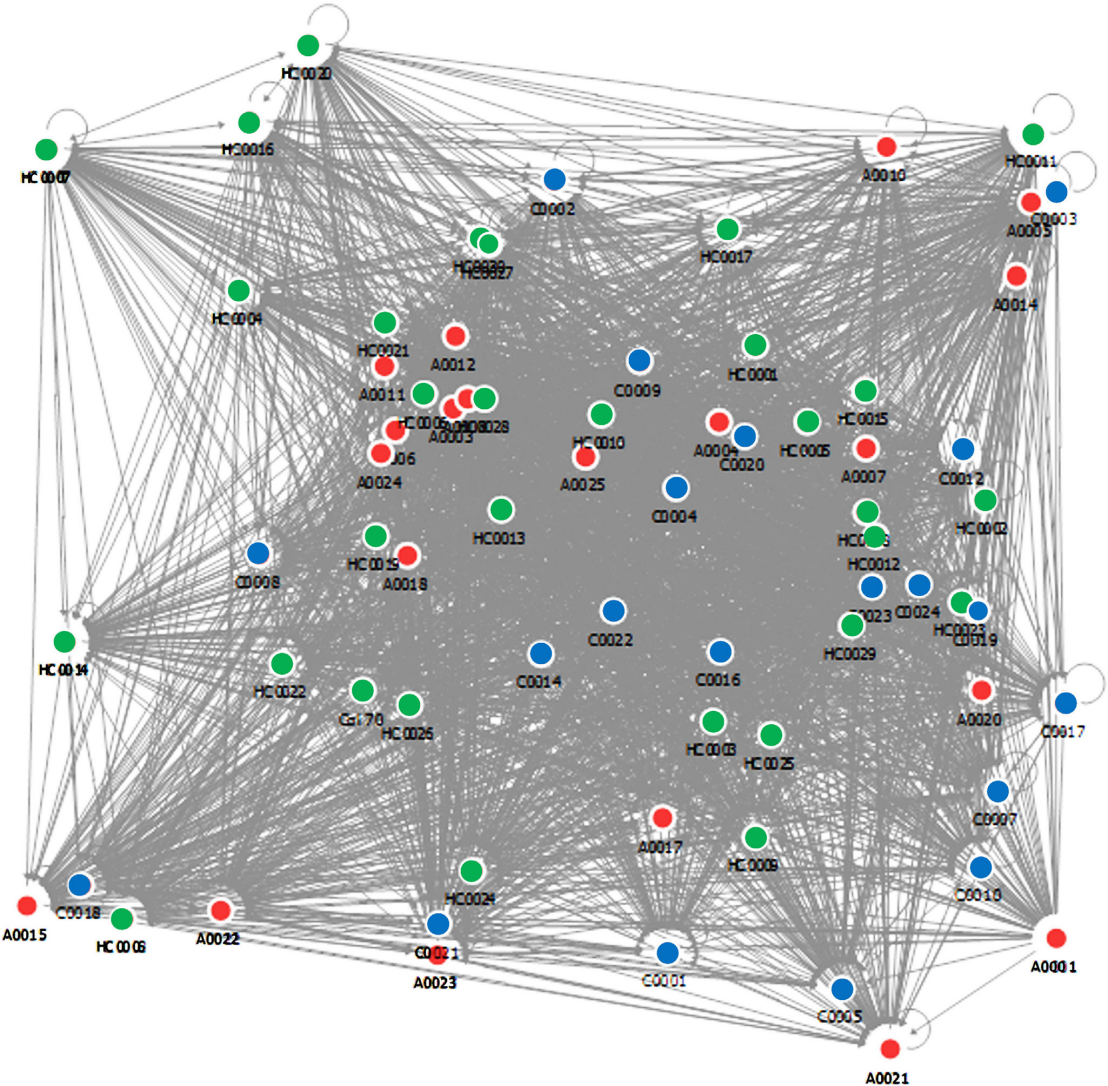
Vector diagram of the steroid ratio relationships.

The database vectors are matched with the vectors of unclassified samples in order to obtain a diagnosis. The algorithm then provides for each subject the steroid ratio relationships with respect to the others, and a complex relational map is obtained by using this approach (Figure 4). The discriminating power is not given by the position of the nodes but from the density of their relationships that is measured in a normalized scale between 1 and 100.

Two database search profile match models were employed to predict the sample classification as control, PCa, or benign prostate hyperplasia. A cartoon of the classical approach and the ratio approach used in the matches are shown in Figure S2 in Supplementary Material. In the case of steroid ratio concentration match, the angular difference among the vectors depends on the steroid concentration ratios, in the case of singular absolute concentration, it is correlated with the absolute concentration only.

We excluded each one of the samples and the learning machine used the other samples as a database to obtain the vectors. Then the sample that was excluded was categorized based on the vectors produced (Table 1). The average output of this learning machine is given in Table 1. There is a clear and significant separation of the PCa (18/20, 90%, all the false negatives were classified as HC), BPH (16/20, 80%, false positives were all PCa), and HC (25/31, 81%, false positives were all PCa) groups (Table 1; Figure 2). The ROC curves were calculated from these data and the area under the curve (again separating the PCa patients from BPH and HC) was 0.8353. The better performance was obtained using the steroid ratio relationship match model exhibiting a sensitivity of 90% and a specificity of 84%. In the case of singular absolute concentration match model the sensitivity (51%) and specificity (52%) are lower. There was no correlation with Gleason score or AR receptor status of any of the classification methods.

Another important information available through the steroid ratio concentration match model is that related to the couple of steroids that could lead to the enzyme classification. It is possible to extrapolate that by minimizing the Geometric Chebyshev distance by means of the SANIST technology (35, 39) and considering a ratio relational coefficient higher than 40 in relative value. Therefore, it is possible to correlate the altered steroids concentration ratios with the biological pathways and consequently with the enzyme potentially responsible for the disease. For example, Table S3 in Supplementary Material shows two steroid relationship ratios that can selectively discriminate patients with prostate carcinoma from benign prostate hyperplasia and healthy controls, together with the associated enzyme (P450C17). This fact opens a way to a future prospective in personalized medicine applications that will provide detailed information to the clinical specialist together with the enzymes that can be targets of certain drugs. This information makes it possible to formulate personalized therapies that re-equilibrate the biochemical disequilibrium. To enrich database with the most relevant individual enzymatic pathways to be correlated with the cancer development and progression, an extensive multi-center study on more subjects is necessary and will be the target of future works.

## Conclusion

Here, we provide a method for quantifying accurately 10 steroids in serum and calculate their ratios. We noted that the levels in the serum of PCa patients were lower than that of BPH and healthy controls, in particular testosterone. Based on clinical and experimental data (8–11, 14), this would imply that supplementation of testosterone in aged men might not be harmful. We also provide a novel SANIST data elaboration application to elaborate steroid profile data in PCa patients and to correlate them with known disease in a learning machine approach. It is based on the evaluation of steroids ratio relationships instead of the simple absolute concentrations. Steroids ratio relationships performed better (sensitivity 90%, specificity 84%) than PSA (sensitivity 63%, specificity 59%) or individual steroids concentrations (sensitivity 51%, specificity 52%). Our study has one limitations, a small sample size. However, it opens a new way to provide to the clinic a diagnostic hypothesis based on metabolic profile together with the steroid concentrations and their ratio relationships that eventually indirectly indicate the enzymes involved in endocrine and metabolic disorders.

## Ethics Statement

The institutional review board ethics committee of IRCCS MultiMedica approved the study (Protocol N 10 2 10/2011). All patients enrolled in the study signed informed consent and accordingly to the Helsinki Declaration of 1975 as revised in 2013.

## Author Contributions

AA: designed the study, conceived the experiments, performed the analysis, wrote the manuscript. AB: conceived the experiments, processed the clinical samples, performed the statistical analysis, and wrote the manuscript. BB: processed the clinical samples, managed the clinical database, and performed the statistical analysis. GA: provided clinical support-patients histological classification GP: provided clinical support, histology evaluation. PC: provided clinical samples, clinical data and the clinical support, and wrote the manuscript. LC: contributed to the MS data analysis. MC: provided important chemical-physical contribution for steroid mass spectra data elaboration. SC: conceived the experiments, run the MS, performed the statistical and bioinformatic analysis, wrote the manuscript. DN: conceived the experiments, performed the statistical analysis, and wrote the manuscript. All authors confirmed they have contributed to the intellectual content of this paper and have met the following three requirements: (a) significant contributions to the conception and design, acquisition of data, or analysis and interpretation of data; (b) drafting or revising the article for intellectual content; and (c) final approval of the published article.

## Conflict of Interest Statement

The authors declare that the research was conducted in the absence of any commercial or financial relationships that could be construed as a potential conflict of interest. The reviewer EZ and handling Editor declared their shared affiliation.

## Supplementary Material

Figure S1 Schematic representation for steroid synthesis starting from the precursor cholesterol and the enzymes involved.

Figure S2 Scheme of singular absolute concentration match and the steroids ratio concentration match models.

Table S1 The internal standard concentrations.

Table S2 The calibrate analyte concentrations and the calculated concentrations of both known and unknown samples.

Table S3 Two steroid relationship ratios that can selectively discriminate patients with prostate carcinoma from benign prostate hyperplasia and healthy controls, together with the associated enzyme (P450C17).
