# Supplementary figures and images for: Serum Steroid Ratio Profiles in Prostate Cancer: A New Diagnostic Tool Toward a Personalized Medicine Approach
Source: Front Endocrinol (Lausanne). 2018 Apr 5;9:110. doi: 10.3389/fendo.2018.00110 (PMC5895774; doi:10.3389/fendo.2018.00110)

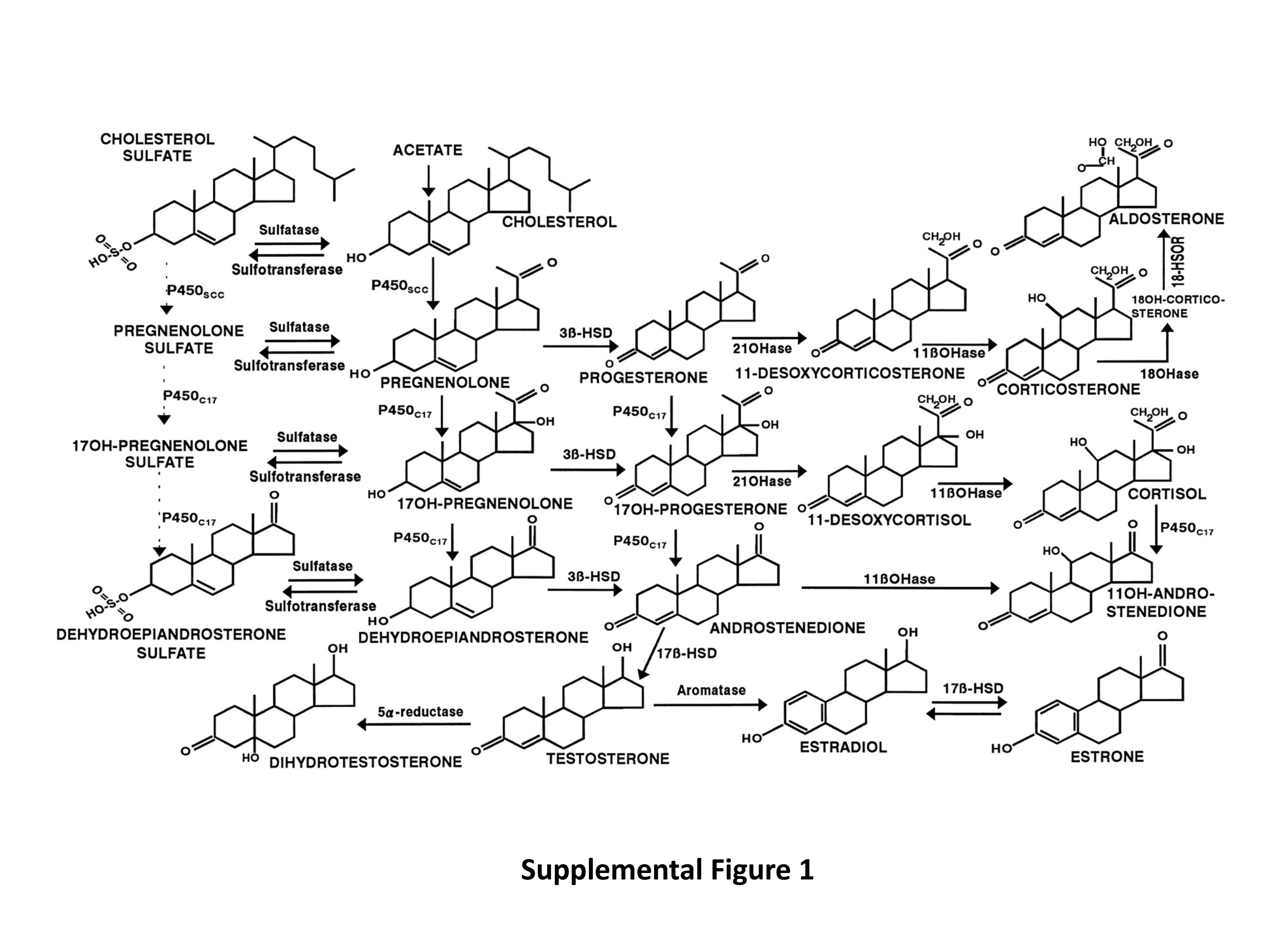

Supplement: Figure S1 — Schematic representation for steroid synthesis starting from the precursor cholesterol and the enzymes involved. [file image_1.jpeg]

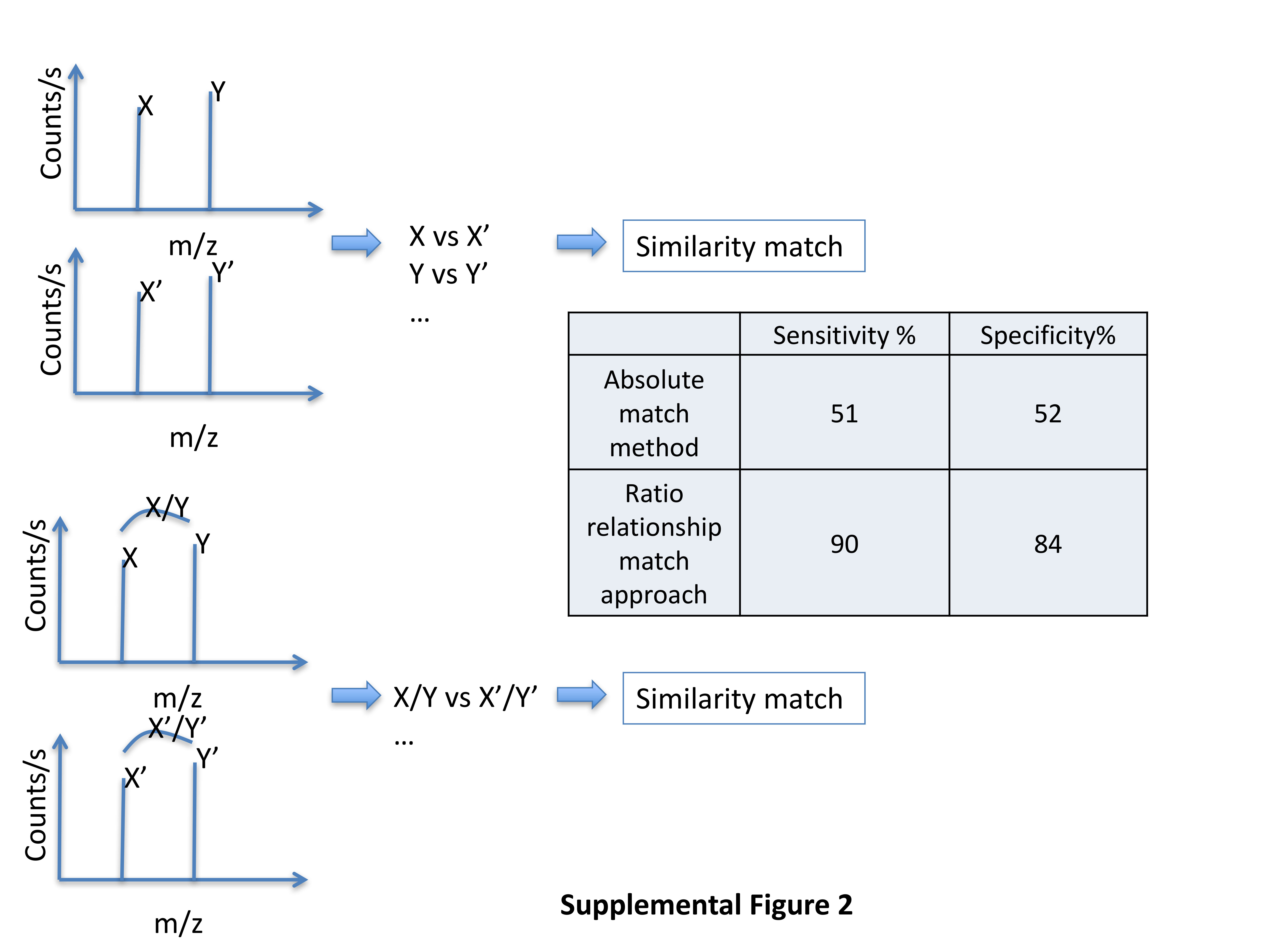

Supplement: Figure S2 — Scheme of singular absolute concentration match and the steroids ratio concentration match models. [file image_2.jpeg]
